# Correction: Immunogenicity and reactogenicity of SARS-CoV-2 vaccines in people living with HIV in the Netherlands: A nationwide prospective cohort study

**DOI:** 10.1371/journal.pmed.1004159

**Published:** 2023-01-06

**Authors:** Kathryn S. Hensley, Marlou J. Jongkees, Daryl Geers, Corine H. GeurtsvanKessel, Yvonne M. Mueller, Virgil A. S. H. Dalm, Grigorios Papageorgiou, Hanka Steggink, Alicja Gorska, Susanne Bogers, Jan G. den Hollander, Wouter F. W. Bierman, Luc B. S. Gelinck, Emile F. Schippers, Heidi S. M. Ammerlaan, Marc van der Valk, Marit G. A. van Vonderen, Corine E. Delsing, Elisabeth H. Gisolf, Anke H. W. Bruns, Fanny N. Lauw, Marvin A. H. Berrevoets, Kim C. E. Sigaloff, Robert Soetekouw, Judith Branger, Quirijn de Mast, Adriana J. J. Lammers, Selwyn H. Lowe, Rory D. de Vries, Peter D. Katsikis, Bart J. A. Rijnders, Kees Brinkman, Anna H. E. Roukens, Casper Rokx

Fig 3 panel B contains a mislabeled axis. The label ‘Pre—HIV negative’ should be ‘Post—HIV negative.’ Please see the correct Fig 3 below.

**Fig 3:**
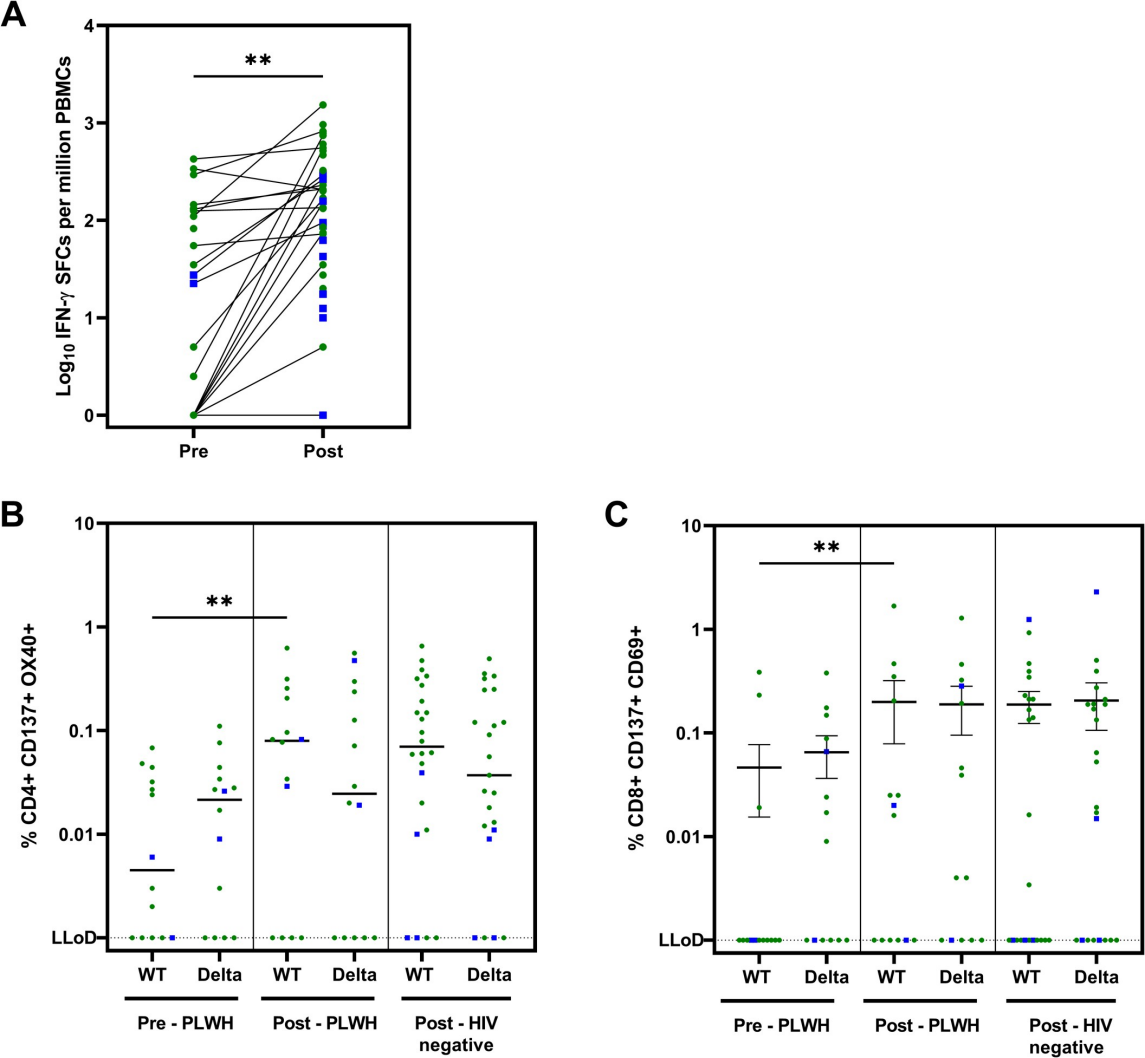
Cellular immune responses against SARS-CoV-2 in subgroup participants (PLWH). (A) Cellular immune response to wild-type spike by ELISpot assay (Pre, *n* = 23; Post, *n* = 45): IFN-γ SFCs after subtraction of MOG. Statistics performed using Mann–Whitney U test: *p* = 0.002. Negative responses: Pre, 8; Post, 5. (B) Cellular immune response to wild-type and Delta spike in AIM assay (*n* = 14): percentage of CD4+ CD137+ OX40+ T-cells after subtraction of DMSO. Dotted line shows the LLoD at 0.001%. Statistics between pre- and post-vaccination for WT in PLWH performed using Wilcoxon matched-pairs signed rank test (*p* = 0.005); pre- and post-vaccination for Delta: ns. Statistics between PLWH and controls with Mann–Whitney U test: not significant. (C) Cellular immune response to wild-type and Delta spike in AIM assay (*n* = 14): percentage of CD8+ CD137+ CD69+ T-cells after subtraction of DMSO. Dotted line shows the LLoD at 0.001%. Statistics performed using Wilcoxon matched-pairs signed rank test (*p* = 0.008); pre- and post-vaccination for Delta: ns. Statistics between PLWH and controls with Mann–Whitney U test: not significant. Green circles: mRNA vaccines; blue squares: vector-based vaccines. Pre: before vaccination; Post: 4–6 weeks after second vaccination. AIM, activation-induced marker; BAU, binding antibody units; cART, combination antiretroviral therapy; DMSO, dimethyl sulfoxide; ELISpot, enzyme-linked immune absorbent spot; GMC, geometric mean concentration; INF-γ, interferon-γ; LLoD, lower limit of detection; MOG, myelin oligodendrocyte glycoprotein; PBMC, peripheral blood mononuclear cell; PLWH, people living with HIV; SFC, spot-forming cell; WT, wild type.
