# Supplementary material for: Immunogenicity and reactogenicity of SARS-CoV-2 vaccines in people living with HIV in the Netherlands: A nationwide prospective cohort study
Source: PLoS Med. 2022 Oct 27;19(10):e1003979. doi: 10.1371/journal.pmed.1003979 (PMC9612532; doi:10.1371/journal.pmed.1003979)
Supplement: S1 Fig — (DOCX) [file pmed.1003979.s001.docx]

**S1 Fig. Gating strategy** **for Flow Cytometry in AIM assay.**

AIM: activation induced marker, MOG: myelin-oligodendrocyte glycoprotein, S: spike

**
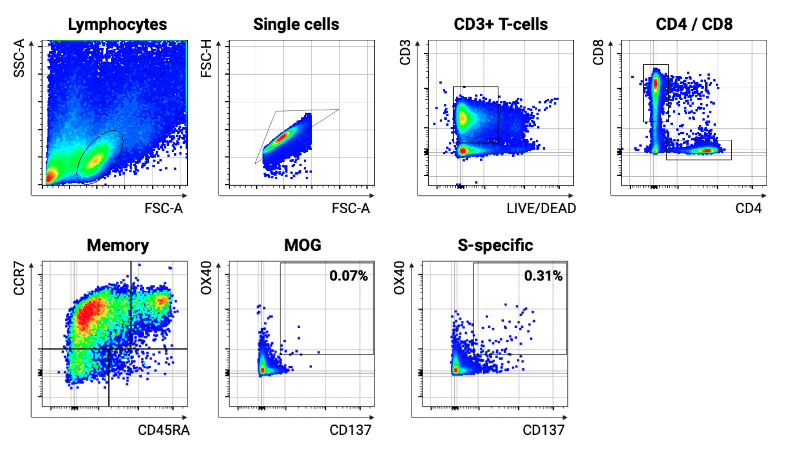
**
