# Supplementary material for: Immunogenicity and reactogenicity of SARS-CoV-2 vaccines in people living with HIV in the Netherlands: A nationwide prospective cohort study
Source: PLoS Med. 2022 Oct 27;19(10):e1003979. doi: 10.1371/journal.pmed.1003979 (PMC9612532; doi:10.1371/journal.pmed.1003979)
Supplement: S2 Fig — (DOCX) [file pmed.1003979.s002.docx]

**S2 Fig. Serologic responses after vaccination in PLWH**

Antibody concentration measured as S-specific binding antibodies per millilitre (BAU/ml) in the subgroup participants (n=43) (Pre is Wantai, all negative). Green circles: PLWH who received mRNA vaccines, Blue squares: PLWH who received vector based vaccines. The dotted line shows the lower limit of detection (4.81 BAU/ml), statistics performed with Wilcoxon matched-pairs signed rank test (**** p<0.001). Pre: before vaccination, Inter: 21 (+/-3) days after first vaccination, Post: 4-6 weeks after second vaccination, S: spike, BAU/ml: binding antibodies per millilitre, PLWH: people living with HIV, LLoD: lower limit of detection

**
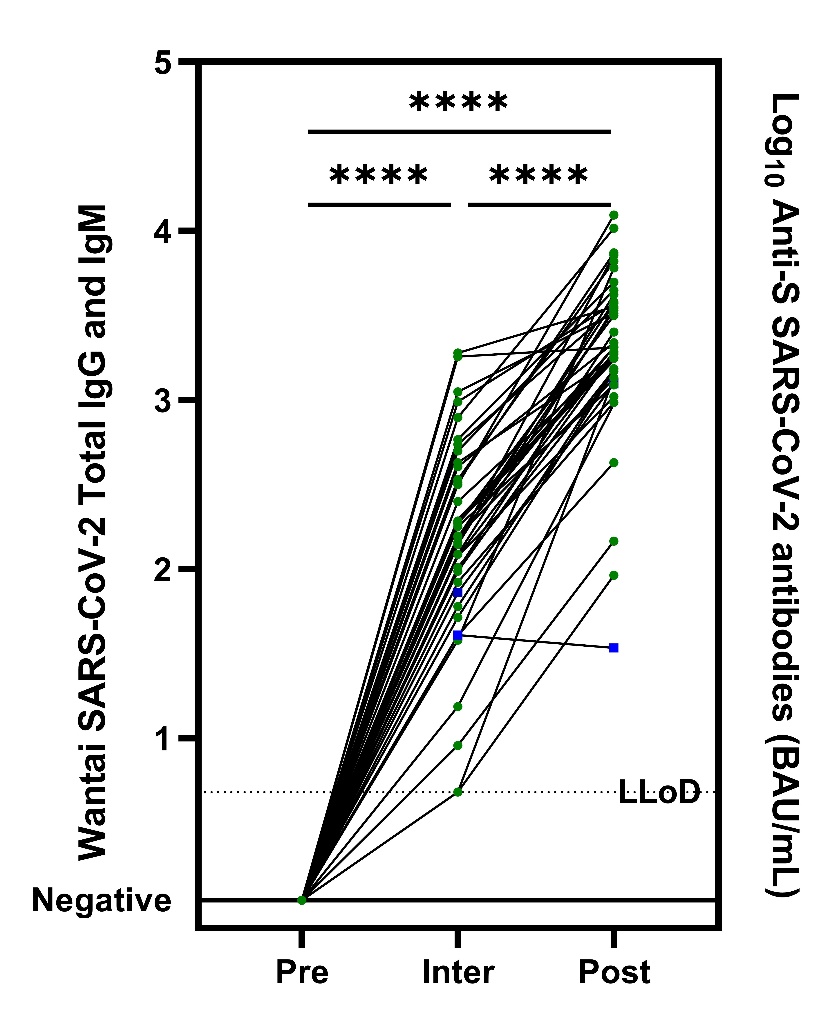
**
