# Supplementary material for: Immunogenicity and reactogenicity of SARS-CoV-2 vaccines in people living with HIV in the Netherlands: A nationwide prospective cohort study
Source: PLoS Med. 2022 Oct 27;19(10):e1003979. doi: 10.1371/journal.pmed.1003979 (PMC9612532; doi:10.1371/journal.pmed.1003979)
Supplement: S3 Fig — (DOCX) [file pmed.1003979.s003.docx]

**S3 Fig: Cellular immune responses against SARS-CoV-2 in subgroup participants (PLWH).**

**
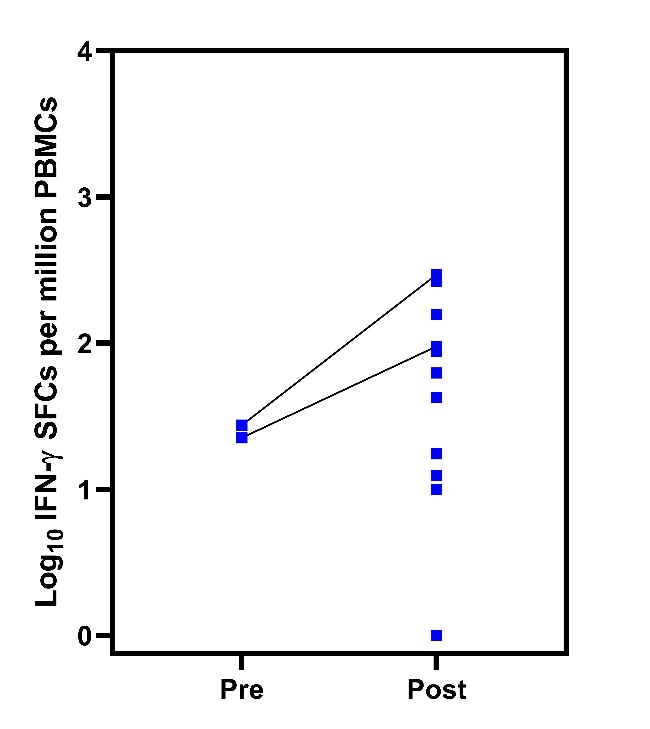

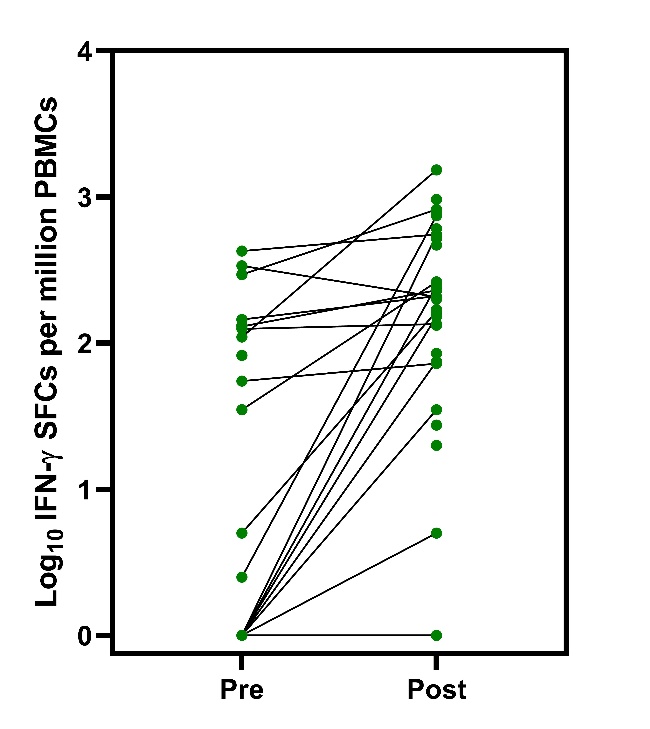
A** Cellular immune response to wild type spike by ELISpot assay (Pre n=21, Post n=3445), IFN-γ SFC after subtraction of MOG in PLWH receiving an mRNA vaccine
**B** Cellular immune response to wild type spike by ELISpot assay (Pre n=2, Post n=11), IFN-γ SFC after subtraction of MOG in PLWH receiving vector vaccine
**C** Cellular immune response to wild type spike by ELISpot assay (Pre n=23, Post n=45), IFN-γ SFC after subtraction of media-DMSO in PLWH. Statistics performed using Mann Whitney test p=0.011
Green circles: mRNA vaccines, Blue squares: vector based vaccines. pre: before vaccination, post: 4-6 weeks after second vaccination, PLWH: people living with HIV, IFN: interferon, SFC: spot forming cells, MOG: myelin- oligodendrocyte glycoprotein, ELISPot: enzyme-linked immune absorbent spot, DMSO: dimethyl sulfoxide

**A B**

**
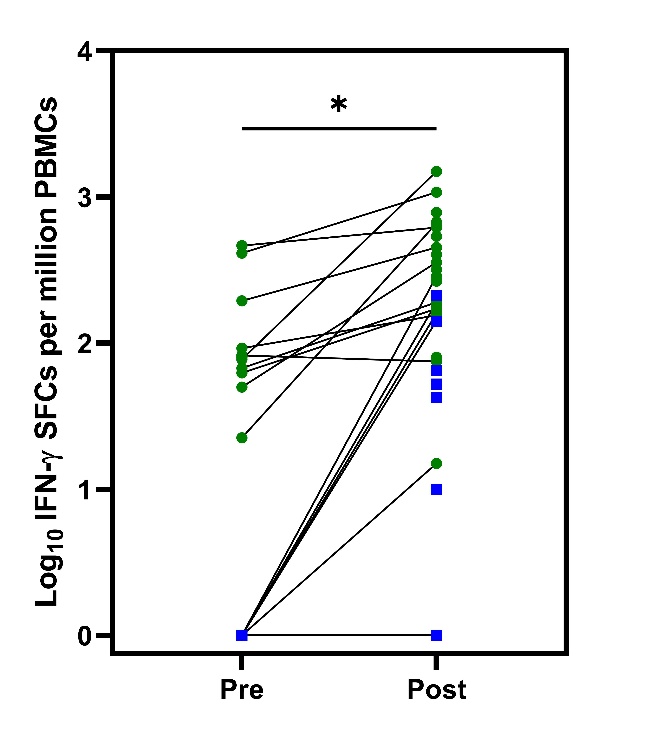
**

**C**
