# Supplementary material for: Immunogenicity and reactogenicity of SARS-CoV-2 vaccines in people living with HIV in the Netherlands: A nationwide prospective cohort study
Source: PLoS Med. 2022 Oct 27;19(10):e1003979. doi: 10.1371/journal.pmed.1003979 (PMC9612532; doi:10.1371/journal.pmed.1003979)

**S4 Fig. ELISpot results in subgroup participants (PLWH)**

All ELISpot assay results shown as IFN-γ SFC per million PBMCs in a log scale, raw data. The thick horizontal bar shows the mean with error bars showing the interquartile range.
Pre: before vaccination, Post: 4-6 weeks after second vaccination, MOG: myelin-oligodendrocyte glycoprotein, SP1: spike protein 1, SP2: spike protein 2, DMSO: dimethyl sulfoxide, NC: nucleocapsid, PLWH: people living with HIV, IFN: interferon, SFC: spot forming cells, PBMCs: peripheral blood mononuclear cells, ELISpot: enzyme-linked immune absorbent spot


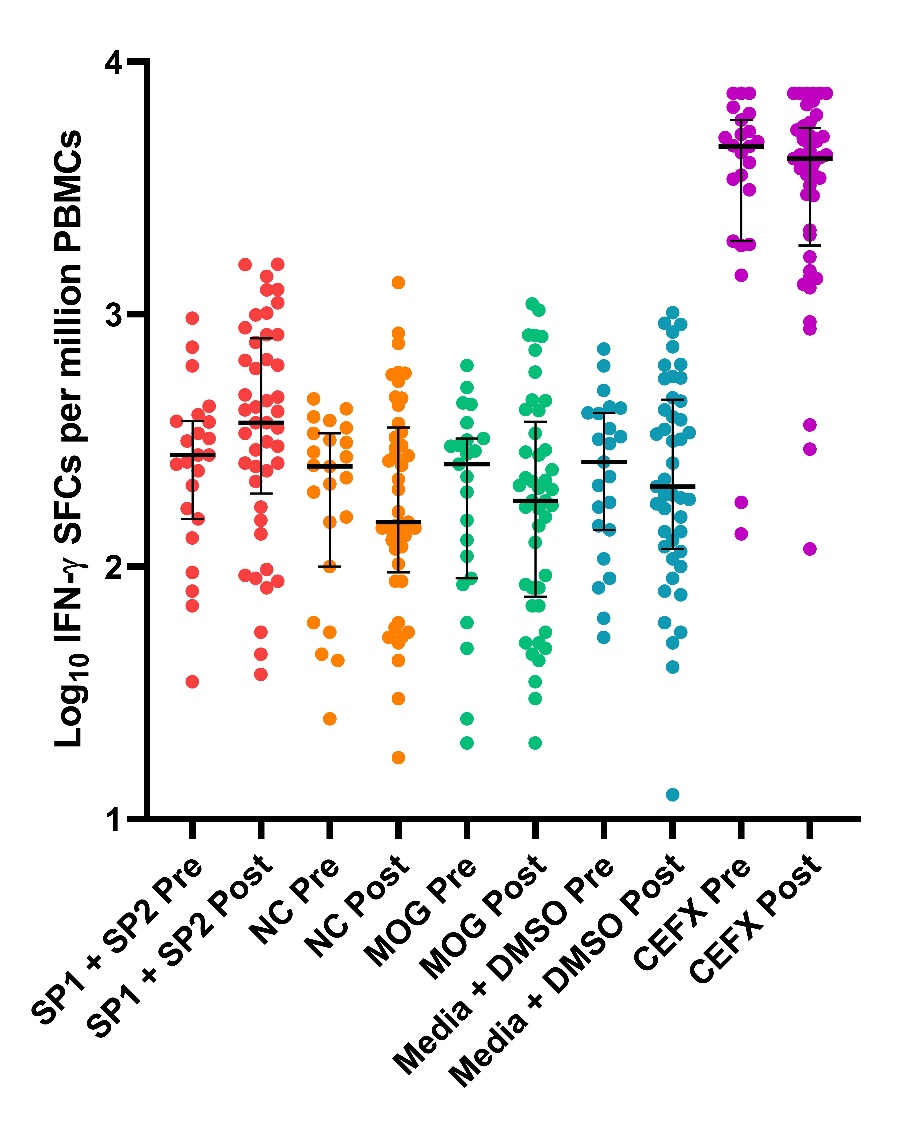

Supplement: S4 Fig — (DOCX) [file pmed.1003979.s004.docx]
