# Supplementary material for: Immunogenicity and reactogenicity of SARS-CoV-2 vaccines in people living with HIV in the Netherlands: A nationwide prospective cohort study
Source: PLoS Med. 2022 Oct 27;19(10):e1003979. doi: 10.1371/journal.pmed.1003979 (PMC9612532; doi:10.1371/journal.pmed.1003979)
Supplement: S1 Table — (DOCX) [file pmed.1003979.s006.docx]

| **S1 Table. Baseline characteristics of HIV negative participants and PLWH per vaccine.** | | | | | | | | | | | |
| --- | --- | --- | --- | --- | --- | --- | --- | --- | --- | --- | --- |
|  | | **HIV negative** | | | | **People living with HIV** | | | | | |
|  |  | **Overall**  N=440 | **BNT162b2**  N=94 (21.4%) | **mRNA-1273**  N=247 (56.1%) | **ChAdOx1-S**  N=26 (5.9%) | **Ad26.COV2.S**  N=73 (16.6%) | **Overall**  N=1154 | **BNT162b2**  N=884 (76.6%) | **mRNA-1273**  N= 100 (8.7%) | **ChAdOx1-S** N=150 (13.0%) | **Ad26.COV2.S**  N=20 (1.7%) |
| **Sex assigned at birth** | | | | | | | | | | | |
|  | Male | 126 (28.6%) | 31 (33%) | 73 (29.6%) | 9 (34.6%) | 13 (17.8%) | 987 (85.5%) | 751 (85.0%) | 88 (88.0%) | 133 (88.7%) | 15 (75.0%) |
|  | Female | 314 (71.4%) | 63 (67.0%) | 174 (70.4%) | 17 (65.4%) | 60 (82.2%) | 167 (14.5%) | 133 (15.0%) | 12 (12.0%) | 17 (11.3%) | 5 (25.0%) |
| **Age category** | | | | | | | | | | | |
|  | 18-55 yr. | 352 (80.0%) | 81 (86.2%) | 203 (82.2%) | 1 (3.8%) | 67 (91.8%) | 703 (60.9%) | 590 (66.7%) | 84 (84.0%) | 12 (8.0%) | 17 (85.0%) |
|  | 56-65 yr. | 74 (16.8) | 13 (13.8%) | 30 (12.1%) | 25 (96.2%) | 9 (8.2%) | 291 (25.2%) | 141 (16.0%) | 16 (16.0%) | 131 (87.3%) | 3 (15.0%) |
|  | 65+ yr. | 14 (3.2%) | 0 | 14 (5.7%) | 0 | 0 | 160 (13.9%) | 153 (17.3%) | 0 | 7 (4.7%) | 0 |
| **On combination antiretroviral therapy** | | | | | | | | | | | |
|  | Yes | NA | NA | NA | NA | NA | 1142 (99.0%) | 873 (98.8%) | 99 (99.0%) | 150 (100%) | 20 (100%) |
|  | No | NA | NA | NA | NA | NA | 12 (1.0%) | 11 (1.2%) | 1 (1.0%) | 0 | 0 |
| **Most recent plasma HIV viral load** | | | | | | | | | | | |
|  | <50 copies/mL | NA | NA | NA | NA | NA | 1127 (97.7%) | 862 (97.5%) | 98 (98.0%) | 147 (98.0%) | 20 (100%) |
|  | ≥50 copies/mL | NA | NA | NA | NA | NA | 26 (2.3%) | 22 (2.5%) | 2 (2.0%) | 3 (2.0%) | 0 |
| **Most recent CD4+ T-cell count** | | | | | | | | | | | |
|  | <250 cells/µL | NA | NA | NA | NA | NA | 41 (3.6%) | 32 (3.6%) | 3 (3.0%) | 5 (3.3%) | 1 (5.0%) |
|  | 250-500 cells/µL | NA | NA | NA | NA | NA | 224 (19.4%) | 168 (19.0%) | 21 (21.0%) | 33 (22.0%) | 2 (10.0%) |
|  | >500 cells/µL | NA | NA | NA | NA | NA | 889 (77.0%) | 684 (77.4%) | 76 (76.0%) | 112 (74.7%) | 17 (85.0%) |
| **CD4+ T-cell count nadir** | | | | | | | | | | | |
|  | <250 cells/µL | NA | NA | NA | NA | NA | 443 (38.4%) | 331 (37.4%) | 34 (34.0%) | 71 (47.3%) | 7 (35.0%) |
|  | 250-500 cells/µL | NA | NA | NA | NA | NA | 376 (32.6%) | 296 (33.5%) | 34 (34.0%) | 40 (26.7%) | 6 (30.0%) |
|  | >500 cells/µL | NA | NA | NA | NA | NA | 152 (13.2%) | 114 (12.9%) | 19 (19.0%) | 17 (11.3%) | 2 (10.0%) |
|  | Unknown | NA | NA | NA | NA | NA | 183 (15.9%) | 143 (16.2%) | 13 (13.0%) | 22 (14.7%) | 5 (25.0%) |
| **Days between doses** | | 28 (25-28) | 21 (21-23) | 28 (28-28) | 56 (56-70) | NA | 35 (35-36) | 35 (35-36) | 35  (34.75-35) | 71.5  (57-77) | NA |
| **Hyporesponse <**300 BAU/mL | | 82 (18.7%) | 0 | 0 | 10 (38.5%) | 72 (98.6%) | 165 (14.2%) | 59 (6.7%) | 4 (4%) | 82 (54.7%) | 20 (100%) |
| **No response**  <33.8 BAU/mL | | 51 (11.6%) | 0 | 0 | 0 | 18 (24.7%) | 33 (2.9%) | 11 (1.2%) | 2 (2%) | 15 (10%) | 5 (25%) |
| Data are n (%) or median (IQR). NA: not applicable, IQR: interquartile range, BAU/mL: binding antibodies per millilitre | | | | | | | | | | | |
