# Supplementary material for: Immunogenicity and reactogenicity of SARS-CoV-2 vaccines in people living with HIV in the Netherlands: A nationwide prospective cohort study
Source: PLoS Med. 2022 Oct 27;19(10):e1003979. doi: 10.1371/journal.pmed.1003979 (PMC9612532; doi:10.1371/journal.pmed.1003979)
Supplement: S2 Table — (DOCX) [file pmed.1003979.s007.docx]

| **S2 Table. Subgroup patient characteristics.** | | | | | |
| --- | --- | --- | --- | --- | --- |
|  | | **Serology** | **ELISpot*** | **AIM** | |
|  | | People living with HIV N=43 | People living with HIV N=47 | People living with HIV N=14 | HIV negative participants N=24 |
| **Vaccine type** | | | | | |
|  | BNT162b2 | 43 (95.3%) | 31 (66.0%) | 12 (85.7%) | 5 (20.8%) |
|  | mRNA**-**1273 | 0 | 4 (8.5%) | 0 | 15 (62.5%) |
|  | ChAdOx1-S | 2 (4.7%) | 12 (25.5%) | 2 (14.3%) | 4 (16.7%) |
|  | Ad26.COV2.S | 0 | 0 | 0 | 0 |
| **Sex assigned at birth** | | | | | |
|  | Male | 52 (77.6%) | 36 (76.6%) | 12 (85.7%) | 5 (20.8%) |
|  | Female | 15 (22.4%) | 11 (23.4%) | 2 (14.3%) | 19 (79.2%) |
| **Age category** | | | | | |
|  | 18-55 yr. | 31 (72.1%) | 26 (55.3%) | 7 (50.0%) | 19 (79.2%) |
|  | 56-65 yr. | 9 (20.9%) | 17 (36.2%) | 5 (35.7%) | 5 (20.8%) |
|  | 65+ yr. | 3 (7.0%) | 4 (8.5%) | 2 (14.3%) | 0 |
| **On combination antiretroviral therapy** | | | | | |
|  | Yes | 42 (97.7%) | 47 (100%) | 14 (100%) | NA |
|  | No | 1 (2.3%) | 0 | 0 | NA |
| **Most recent plasma HIV viral load** | | | | | |
|  | <50 copies/mL | 42 (97.7%) | 47 (100%) | 14 (100%) | NA |
|  | ≥50 copies/mL | 1 (2.3%) | 0 | 0 | NA |
| **Most recent CD4+ T-cell count** | | | | | |
|  | <250 cells/µL | 3 (7.0%) | 3 (6.4%) | 3 (21.4%) | NA |
|  | 250-500 cells/µL | 5 (11.6%) | 6 (12.8%) | 4 (28.6%) | NA |
|  | >500 cells/µL | 35 (81.4%) | 38 (80.9%) | 7 (50.0%) | NA |
| **CD4 T-cell count nadir** | | | | | |
|  | <250 cells/µL | 14 (32.6%) | 18 (38.3%) | 7 (50.0%) | NA |
|  | 250-500 cells/µL | 15 (34.9%) | 14 (29.8%) | 3 (21.4%) | NA |
|  | >500 cells/µL | 7 (16.3%) | 5 (10.6%) | 1 (7.1%) | NA |
|  | Unknown | 7 (16.3%) | 10 (21.3%) | 3 (21.4%) | NA |
| *ELISpot: before vaccination n= 23, after vaccination n=45 Data are n (%) NA: not applicable, IQR: interquartile range, ELISPot: enzyme-linked immune absorbent spot, AIM: activation induced marker | | | | | |
