# Supplementary material for: Immunogenicity and reactogenicity of SARS-CoV-2 vaccines in people living with HIV in the Netherlands: A nationwide prospective cohort study
Source: PLoS Med. 2022 Oct 27;19(10):e1003979. doi: 10.1371/journal.pmed.1003979 (PMC9612532; doi:10.1371/journal.pmed.1003979)
Supplement: S3 Table — (DOCX) [file pmed.1003979.s008.docx]

**S3 Table. Regression model to investigate the difference in antibody concentration between PLWH and HIV-uninfected controls vaccinated with one of the two available mRNA vaccines (BNT162b2 or mRNA-1273).** Back transformed estimated regression coefficients, 95% Confidence intervals and p-values from the multivariable linear regression model for log(antibody after vaccination). The antibody concentration was log-transformed in order to avoid deviations from normality assumptions.

|  | **Estimate (95% CI)** | **P** |
| --- | --- | --- |
| **(Intercept)** | 3068.565 (2599.373; 3622.447) | <0.001 |
| **mRNA-1273** | 1.431 (1.220; 1.679) | <0.001 |
| **HIV-positive** | 0.607 (0.508; 0.725) | <0.001 |
| **Male sex assigned at birth** | 0.769 (0.667; 0.888) | <0.001 |
| **Age category 56-65** | 0.902 (0.772; 1.054) | 0.194 |
| **Age category 65+** | 0.645 (0.544; 0.765) | <0.001 |

PLWH: people living with HIV, CI: confidence interval
