# Supplementary material for: Immunogenicity and reactogenicity of SARS-CoV-2 vaccines in people living with HIV in the Netherlands: A nationwide prospective cohort study
Source: PLoS Med. 2022 Oct 27;19(10):e1003979. doi: 10.1371/journal.pmed.1003979 (PMC9612532; doi:10.1371/journal.pmed.1003979)
Supplement: S4 Table — (DOCX) [file pmed.1003979.s009.docx]

**S4 Table. HIV-related and HIV-unrelated factors associated with the height of antibody response after vaccination with one of the two available mRNA vaccines (BNT162b2 or mRNA-1273) in PLWH.** Back transformed estimated regression coefficients, 95% Confidence intervals and p-values from the multivariable linear regression model for log(antibody concentration after vaccination). The antibody concentration was log-transformed in order to avoid deviations from normality assumptions.

|  | **Estimate (95% CI)** | **P** |
| --- | --- | --- |
| **(Intercept)** | 690.871 (450.594; 1059.277) | <0.001 |
| **mRNA-1273** | 1.572 (1.225; 2.018) | <0.001 |
| **Male sex assigned at birth** | 0.693 (0.555; 0.865) | 0.001 |
| **Age category 56-65** | 0.913 (0.741; 1.126) | 0.396 |
| **Age category 65+** | 0.654 (0.526; 0.814) | <0.001 |
| **Viral load >50 copies/mL** | 0.454 (0.286; 0.720) | 0.001 |
| **CD4 nadir 250-500 cells/µL** | 1.069 (0.897; 1.274) | 0.456 |
| **CD4 nadir > 500 cells/µL** | 0.984 (0.776; 1.249) | 0.895 |
| **CD4 250-500 cells/µL** | 2.845 (1.876; 4.314) | <0.001 |
| **CD4 > 500 cells/µL** | 2.936 (1.961; 4.394) | <0.001 |

PLWH: People living with HIV, CI: confidence interval
