# Supplementary material for: Immunogenicity and reactogenicity of SARS-CoV-2 vaccines in people living with HIV in the Netherlands: A nationwide prospective cohort study
Source: PLoS Med. 2022 Oct 27;19(10):e1003979. doi: 10.1371/journal.pmed.1003979 (PMC9612532; doi:10.1371/journal.pmed.1003979)
Supplement: S5 Table — (DOCX) [file pmed.1003979.s010.docx]

**S5 Table. Regression model to investigate the difference in antibody concentration between PLWH and HIV-uninfected controls vaccinated with one of the two vector vaccines (AZD1222 and Ad26.COV2.S).** Back transformed estimated regression coefficients, 95% Confidence intervals and p-values from the multivariable linear regression model for log(antibody after vaccination). The antibody concentration was log-transformed in order to avoid deviations from normality assumptions.

|  | **Estimate (95% CI)** | **P** |
| --- | --- | --- |
| **(Intercept)** | 519.394 (278.916; 967.212) | <0.001 |
| **Ad26.COV2.S** | 0.118 (0.065; 0.212) | <0.001 |
| **HIV positive** | 0.605 (0.387; 0.945) | 0.027 |
| **Male sex assigned at birth** | 1.145 (0.766; 1.711) | 0.508 |
| **Age category 56-65** | 0.676 (0.397; 1.152) | 0.149 |
| **Age category 65+** | 0.486(0.171; 1.382) | 0.175 |

PLWH: people living with HIV, CI: confidence interval
