# Supplementary material for: Immunogenicity and reactogenicity of SARS-CoV-2 vaccines in people living with HIV in the Netherlands: A nationwide prospective cohort study
Source: PLoS Med. 2022 Oct 27;19(10):e1003979. doi: 10.1371/journal.pmed.1003979 (PMC9612532; doi:10.1371/journal.pmed.1003979)
Supplement: S6 Table — (DOCX) [file pmed.1003979.s011.docx]

**S6 Table. HIV-related and HIV-unrelated factors associated with the height of antibody response after vaccination with one of the two vector vaccines (AZD1222 and Ad26.COV2.S) in PLWH.** Back transformed estimated regression coefficients, 95% Confidence intervals and p-values from the multivariable linear regression model for log(antibody after vaccination). The antibody concentration was log-transformed in order to avoid deviations from normality assumptions.

|  | **Estimate (95% CI)** | **P** |
| --- | --- | --- |
| **(Intercept)** | 44.711 (10.663; 187.476) | <0.001 |
| **Ad26.COV2.S** | 0.123 (0.051; 0.300) | <0.001 |
| **Male sex assigned at birth** | 1.410 (0.723; 2.749) | 0.311 |
| **Age category 56-65** | 0.493 (0.227; 1.075) | 0.075 |
| **Age category 65+** | 0.472 (0.117; 1.899) | 0.288 |
| **Viral load >50 copies/mL** | 0.357 (0.053; 2.404) | 0.287 |
| **CD4 nadir 250-500 cells/µL** | 0.374 (0.637; 1.086) | 0.097 |
| **CD4 nadir > 500 cells/µL** | 0.642 (0.318; 1.296) | 0.214 |
| **CD4 250-500 cells/µL** | 7.400 (2.278; 24.035) | 0.001 |
| **CD4 > 500 cells/µL** | 11.180 (3.533; 35.372) | <0.001 |

PLWH: People living with HIV, CI: confidence interval
