# Supplementary material for: Immunogenicity and reactogenicity of SARS-CoV-2 vaccines in people living with HIV in the Netherlands: A nationwide prospective cohort study
Source: PLoS Med. 2022 Oct 27;19(10):e1003979. doi: 10.1371/journal.pmed.1003979 (PMC9612532; doi:10.1371/journal.pmed.1003979)
Supplement: S7 Table — (DOCX) [file pmed.1003979.s012.docx]

**S7 Table. Linear regression model to investigate factors associated with the antibody response after completion of the vaccination schedule in PLWH with antibody concentration above the minimal level of clinical protection (≥300 BAU/mL).** Estimated odds ratios, 95% Confidence intervals and p-values from the multivariable linear regression model for or antibody concentration above or below 300 BAU/mL.

|  | **OR Estimate (95% CI)** | **P** |
| --- | --- | --- |
| **(Intercept)** | 3.454 (1.363; 9.297) | 0.011 |
| **Vector vaccine type** | 0.036 (0.019; 0.064) | <0.001 |
| **Male sex assigned at birth** | 0.867 (0.434; 1.651) | 0.674 |
| **Age category 56-65** | 0.832 (0.454; 1.576) | 0.562 |
| **Age category 65+** | 0.282 (0.152; 0.529) | <0.001 |
| **Viral load >50 copies/mL** | 0.266 (0.094; 0.841) | 0.017 |
| **CD4 nadir 250-500 cells/µL** | 0.740 (0.439; 1.241) | 0.255 |
| **CD4 nadir > 500 cells/µL** | 1.012 (0.492; 2.144) | 0.974 |
| **CD4 250-500 cells/µL** | 8.143 (3.294; 20.080) | <0.001 |
| **CD4 > 500 cells/µL** | 9.177 (3.837; 21.715) | <0.001 |

PLWH: People living with HIV OR: odds ratio, CI: confidence interval
