# Supplementary material for: Immunogenicity and reactogenicity of SARS-CoV-2 vaccines in people living with HIV in the Netherlands: A nationwide prospective cohort study
Source: PLoS Med. 2022 Oct 27;19(10):e1003979. doi: 10.1371/journal.pmed.1003979 (PMC9612532; doi:10.1371/journal.pmed.1003979)
Supplement: S8 Table — (DOCX) [file pmed.1003979.s013.docx]

**S8 Table. Linear regression model to investigate factors associated with the antibody response after completion of the vaccination schedule in PLWH with a quantifiable antibody concentration (≥33.8 BAU/mL).** Estimated odds ratio, 95% Confidence intervals and p-values from the multivariable linear regression model for antibody concentration above or below 33.8 BAU/mL.

|  | **OR Estimate (95% CI)** | **P** |
| --- | --- | --- |
| **(Intercept)** | 7.954 (2.212; 36.137) | 0.003 |
| **Vector vaccine type** | 0.029 (0.011; 0.074) | <0.001 |
| **Male sex assigned at birth** | 0.648 (0.177; 1.835 ) | 0.457 |
| **Age category 56-65** | 3.697 (1.412; 9.987) | 0.009 |
| **Age category 65+** | 1.487 (0.480; 5.763) | 0.523 |
| **Viral load >50 copies/mL** | 0.298 (0.078; 1.392) | 0.094 |
| **CD4 nadir 250-500 cells/µL** | 0.722 (0.303; 1.724) | 0.460 |
| **CD4 nadir > 500 cells/µL** | 1.449 (0.404; 6.940) | 0.598 |
| **CD4 250-500 cells/µL** | 7.573 (2.431; 23.841) | <0.001 |
| **CD4 > 500 cells/µL** | 16.894 (5.198; 55.956) | <0.001 |

PLWH: People living with HIV OR: odds ratio, CI: confidence interval
