# Supplementary material for: Immunogenicity and reactogenicity of SARS-CoV-2 vaccines in people living with HIV in the Netherlands: A nationwide prospective cohort study
Source: PLoS Med. 2022 Oct 27;19(10):e1003979. doi: 10.1371/journal.pmed.1003979 (PMC9612532; doi:10.1371/journal.pmed.1003979)
Supplement: S1 Text — (DOCX) [file pmed.1003979.s014.docx]

**S1 Text. Additional information on study design and participants.**

All adult inhabitants of the Netherlands received an invitation for voluntary first round of vaccination with either BNT162b2, mRNA-1273, ChAdOx1-S (AZD1222), or Ad26.COV2.S between January and August 2021. The type of vaccination offered in the Netherlands was decided by the National Institute for Public Health and the Environment and was dependent on vaccine availability, employment in healthcare, age, and comorbidities. The government started vaccinating all healthcare workers and people above 65 years old with BNT126b2 from the end of January 2021. Subsequently, from the middle of February, all people between 60 and 64 years were invited for vaccination with ChAdOx1-S. From March onwards, all people with a medical indication, which included PLWH, between 18 and 60 years old were invited for vaccination with either BNT162b2, ChAdOx1-S or mRNA-1273. However, on the 8th of April the Governmental Dutch Health council suspended vaccination with ChAdOx1-S in people under the age of 60 due to the possibility of blood clots with low levels of platelets. The interval between the BNT162b2 vaccines was six weeks and between the mRNA-1273 vaccines five weeks. Up to 20 May 2021 the interval between the ChAdOx1-S vaccines was 12 weeks, after which the interval was changed to between four and 12 weeks. The Ad26.COV2.S vaccine was administered only once.
