# Supplementary material for: Immunogenicity and reactogenicity of SARS-CoV-2 vaccines in people living with HIV in the Netherlands: A nationwide prospective cohort study
Source: PLoS Med. 2022 Oct 27;19(10):e1003979. doi: 10.1371/journal.pmed.1003979 (PMC9612532; doi:10.1371/journal.pmed.1003979)
Supplement: S2 Text — (DOCX) [file pmed.1003979.s015.docx]

**S2 Text. Additional information on ELISpot assay.**

ELISpot assays were performed on cryopreserved PBMCs using a commercial double colour kit (Cellular Technology Limited). ImmunoSpot plates were coated with capture antibodies and kept overnight at 4°C. The plates were washed one of the following overlapping peptide pools were added as stimuli to the appropriate wells, with culture media (CTL-Test medium with 1% L-Glutamine): spike (S) glycoprotein (PM-WCPV-S1), nucleocapsid (NC) protein (PM-WCPV-NCAP-1), myelin-oligodendrocyte glycoprotein (MOG) (PM-MOG, negative control), and CEFX (PM-CEFX-2, positive control) (all from JPT) at a concentration of 2 µg/ml. PBMCs were plated at 200,000 cells per well and incubated 72 hours (9% CO_2_). Cells were removed, detection antibodies and enzymes for visualisation of the cytokines were added, after which plates were air dried in the dark prior to counting with the ImmunoSpot Image Analyzer.
