# Supplementary material for: Immunogenicity and reactogenicity of SARS-CoV-2 vaccines in people living with HIV in the Netherlands: A nationwide prospective cohort study
Source: PLoS Med. 2022 Oct 27;19(10):e1003979. doi: 10.1371/journal.pmed.1003979 (PMC9612532; doi:10.1371/journal.pmed.1003979)
Supplement: S3 Text — (DOCX) [file pmed.1003979.s016.docx]

**S3 Text. Additional information on AIM assay.**

Activation induced marker (AIM) assays were also performed on cryopreserved PBMCs. 1x10^6^ PBMCs were incubated with SARS-CoV-2 peptide pools (1µg/mL) and incubated at 37°C for 20 hours. SARS-CoV-2 peptide pools consist of 15-mers with 10 amino acid overlaps covering the entire S-protein of the WuhanHu1 (wild-type) (in PLWH) and D614G (wild-type) (in HIV-negative controls) or B.1.617.2 (delta) variant. PBMCs were stimulated with an equimolar amount dimethyl sulfoxide (DMSO) as negative control or a combination of PMA (50 ng/mL) and Ionomycin (500 ng/mL) as positive control. Following stimulation, cells were stained and measured by flow cytometry (FACSLyric, BD). PBMC were stained at 4°C for 15 minutes with the following antibodies and dilutions: anti-CD3^PerCP^ (Clone SK7, BD, 1:25), anti-CD4^V50^ (Clone L200, BD, 1:50), anti-CD8^FITC^ (Clone DK25, Dako, 1:25), anti-CD45RA^PE-Cy7^ (Clone L48, BD, 1:50), anti-CCR7^BV711^, anti-CD69^APC-H7^ (Clone FN50, BD, 1:50), anti-CD137^PE^ (Clone 4B4-1, Miltenyi, 1:50), and anti-OX40^BV605^ (Clone L106, BD, 1:25). LIVE/DEAD™ Fixable Aqua Dead Cell staining was included (AmCyan, Invitrogen, 1:100).
