# Supplementary material for: Immunogenicity and reactogenicity of SARS-CoV-2 vaccines in people living with HIV in the Netherlands: A nationwide prospective cohort study
Source: PLoS Med. 2022 Oct 27;19(10):e1003979. doi: 10.1371/journal.pmed.1003979 (PMC9612532; doi:10.1371/journal.pmed.1003979)
Supplement: Attachment — Submitted filename: Response to reviewers.docx [file pmed.1003979.s017.docx]

Rotterdam, 22 June 2022

Dear Dr. Turner, Dr. Bosurgi,

We thank the editorial board of PLoS Medicine and the specialist reviewers for their valuable suggestions and time to review our work during these unprecedented times. Please find below the point-by-point replies to the questions that were raised, with our answers in italics and the location of the changes in the marked-up copy of the manuscript underlined.

**Comments from Academic editor**

I would have preferred to see data presented on neutralizing antibodies as well since that is regarded as the major mediator of protection. However, the authors argument that neutralizing Abs have been shown to correlate with IgG titres is a reasonable point. As an alternative, the authors could consider including data generated using a surrogate ELISA-based neutralization assay which is available commercially and suitable for testing large number of samples without specialised culture facilities. I would also like to have seen a little more analysis of differences in Abs between HIV+ vs HIV-, such as IgG subclasses which are relevant in immunity and can be impacted by HIV.

*We did not perform a neutralisation assay; we have now elaborated on the reasons for not performing this assay to the manuscript and the evidence this was based on. This is mainly because neutralisation correlated with magnitude of antibody response. Whilst we believe that this could indeed be of additional value, considering the reasons given, and the time and manpower necessary to perform these tests, and also the importance of our message to be released as soon as possible, we have not performed any additional tests.*

**Requests from the editors:**

1)	Please revise your title: Please place the study setting before the colon. For example, “… in the Netherlands: a nationwide prospective cohort study”

*The title has been changed.* Page 1, line 2

2)	The Data Availability Statement (DAS) requires revision. For each data source used in your study:

a) If the data are freely or publicly available, note this and state the location of the data: within the paper, in Supporting Information files, or in a public repository (include the DOI or accession number).

b) If the data are owned by a third party but freely available upon request, please note this and state the owner of the data set and contact information for data requests (web or email address). Note that a study author cannot be the contact person for the data.

c) If the data are not freely available, please describe briefly the ethical, legal, or contractual restriction that prevents you from sharing it. Please also include an appropriate contact (web or email address) for inquiries (again, this cannot be a study author).

*The data is available upon request. Statement has been added.* Page 3, line 57-60

3)	Abstract:

a)	Abstract Background: The final sentence should clearly state the study question.

*The study question has been added.* Page 4, line 63-64

b)	Please ensure that all numbers presented in the abstract are present and identical to numbers presented in the main manuscript text.

*Numbers in abstract and text have been checked.* Page 4 and 5

c)	Please include the actual amounts and/or absolute risk(s) of relevant outcomes

d)	Please quantify the main results (please present both 95% CIs and p values).

*p-values have been added.* Page 4 and 5

e)	In the last sentence of the Abstract Methods and Findings section, please describe the main limitation(s) of the study's methodology.

*The largest limitation of the study has now been added to the abstract.* Page 5 line 88-89

4)	Author summary - At this stage, we ask that you reformat your non-technical Author Summary. The Author Summary should immediately follow the Abstract in your revised manuscript. This text is subject to editorial change and should be distinct from the scientific abstract. The summary should be accessible to a wide audience that includes both scientists and non-scientists.

*An author summary has been added after the abstract,* Page 5 and 6, lines 96-126

5)	Please conclude the Introduction with a clear description of the study question or hypothesis.

*The introduction has been elaborated to more clearly include the hypothesis and study question.* Page 8, lines 155-159

6)	Methods:

a)	Please report the number of patients and samples, and dates of recruitment

b)	Please report how many patients were "lost to follow-up" as used in this study.

*The number of particpants included and lost to follow up are mentioned in the results section of the manuscript, but have been added to the methods.* Page 10, line 177-181

7)	Please add the following statement, or similar, to the Methods: "This study is reported as per the Strengthening the Reporting of Observational Studies in Epidemiology (STROBE) guideline (S1 Checklist)."

*This has been added.* Page 9, line 174 and 175

a)	Thank you for providing the STROBE checklist. When completing the checklist, please use section and paragraph numbers, rather than page numbers.

*The checklist has been added and filled in.* Supplemental data

8)	Please provide p values in addition to 95% CIs in the main text and tables

*p-values have been added where they were missing.* Page 16, line 314

9)	Please indicate in the figure captions the meaning of the bars and whiskers in figure 2, S3

*The definition has been added to the figure legend.* Captions of figures 2 and S3

10)	Please use up to 3 decimal points for p-values

*All p-values have been changed to 3 decimal points.* Page 18, line 259, 366 and 367

11)	Please define all abbreviations used in footnotes of tables and figures

*The abbreviations have been checked and added where missing.*

12)	Please replace "subject" with participant, patient, individual, or person.

*‘Subject’ has been changed to ‘person’.* Page 9, line 164

13)	Please replace “HIV patients” with “people living with HIV”

*“chronic HIV patients” has been replaced with “people living with chronic HIV”.* Page 24, line 444

14)	Please re-label the Conclusion section to Discussion.

*This has also been changed in the abstract.* Page 5, line 90

15)	Please organize the Discussion as follows: a short, clear summary of the article's findings; what the study adds to existing research and where and why the results may differ from previous research; strengths and limitations of the study; implications and next steps for research, clinical practice, and/or public policy; one-paragraph conclusion.

*We have organized the discussion:
a short, clear summary of the article's findings: paragraph 1
what the study adds to existing research and where and why the results may differ from previous research: paragraph 2-4
strengths and limitations of the study: paragraph 5 and 6
implications and next steps for research, clinical practice, and/or public policy: paragraph 7
one-paragraph conclusion: paragraph 8*

16)	References: Please include access dates for all weblinks and ensure that all weblinks are current and accessible e.g ref # 28

*This has been updated.*

17)	We suggest you copyedit your manuscript for language usage, spelling, and grammar.

*We have copyedited the manuscript*

**Comments from the reviewers:**

**Reviewer #1:**

This prospective cohort study aims to examine the immunogenicity of BNT162b2, mRNA-1273, ChAdOx1-S and 57 Ad26.COV2.S vaccines in adult PLWH, without prior COVID-19, compared to HIV-negative controls.

Comments:

"Between February-September 2021, 1154 PLWH (median age 53 [IQR 44-60], 86% male) and 440 controls (median age 43 [IQR 33-53], 29% male) were included."

And

"We performed a prospective observational cohort study in 22 of the 24 HIV treatment centres in the Netherlands."

And

"Inclusion was stratified and monitored to best represent represents the Dutch population of PLWH (S1 appendix)"

Can the authors please provide further information on this stratification process? Did the authors consider including any stratification variables within the analysis?

*The requested information on the stratification has been moved from the appendix to the main manuscript.*

*The stratification variables were used in the analysis.* Page 9, line 167-171

Additionally, can the authors comment on whether the stratification and monitoring they completed was successful (i.e. do the authors consider the analysed sample to be representative of the wider population)?

*This text has been elaborated in the discussion.* Page 24, line 453-454

"Blood samples were collected up to six weeks before vaccination (T0) and four to six weeks after the completed vaccination schedule (T2)."

Did the authors consider including time to sample collection (after completed vaccination schedule) as a covariate in the analysis?

*We have now added to time to sample collection in Table 1. There is no significant difference between the groups and therefore we did not add it as a covariate in the analyses.* Page 15, line 301

"In a subgroup, additional blood samples were collected, 21 days (+/- three days) after the first vaccination for serology (T1) or peripheral blood mononuclear cells (PBMCs) at any of the study visits."

Can the authors please provide further information here? For instance, can the authors please specify in the Methods the number of paricipants included in these subgroups, and how the participants were selected (e.g. randomly)?

*Patients in the subgroup participated if they were willing to participate in extra sampling, this text has been added. Some discussion has been added on the characteristics.* Page 10, line 183-186, Page 25 line 464-4665zats:

"ELISpot T0 to T2 data and AIM data comparing PLWH and controls were analysed by Mann-Whitney-U tests."

S2 table shows these subgroups, and it can be seen that there are no HIV negative controls over 65 years of age. Did the authors consider including covariates in this analysis?

*Considering the small numbers of participants in the sub group analyses, we decided to not include covariates in these analyses*

Can the authors please discuss the small sample sizes included for these subgroup analyses?

*This has been added to the discussion.* Page 24-25, line 463-466

"We justified the sample size by calculating that 556 PLWH receiving mRNA vaccines would be sufficient to detect a serological response rate of 90% or lower compared to a hypothetical 95% response rate in controls with >80% power. When the control group was confirmed, and before the data lock and endpoint analyses, we amended the protocol to update the sample size calculation. Accounting for the imbalance in the number of controls versus PLWH with BNT162b2 and mRNA-1273 vaccinations, we found that 286 controls were sufficient to detect a 20% lower antibody response in PLWH with >80% power and alpha 5%."

The authors suitably provide the basis of the sample size calculations, and associated assessment of study power.

*Thank you*

"Median (interquartile range (IQR)), or n (%) for descriptive data were used. A multivariable linear regression model was used for the analysis of the anti-spike SARS-CoV-2 IgG. The outcome was transformed using the natural logarithm plus one unit: ln(anti-spike SARS-CoV-2 IgG+1) in order to meet the model assumptions. "

and

"In subgroup participants we evaluated differences from baseline to T1 and T1 to T2, as well as AIM data comparing T0 and T2 time points, by Wilcoxon matched-pairs signed rank test."

Technically appropriate statistical techniques and modelling methods have been applied by the authors within the context of this research.

*Thank you*

"The model was further adjusted for differences in vaccine type, age, and sex. A multivariable linear regression model was also used to quantify the difference in ln(anti-spike SARS-CoV-2 IgG+1) between PLWH versus controls for the subset in the sample vaccinated with vector vaccines. A similar model was used to quantify the effect of age, sex, vaccine type, most recent CD4+ T-cell count, and antibody concentration in PLWH. In addition, multivariable logistic regression models were used to calculate odds ratios with 95%CI for the effects of sex, age, nadir CD4+ T-cell count, most recent CD4+ T-cell count, HIV-RNA viral load, and vaccine group in PLWH on having a hyporesponse or a non response."

Do the authors have information on ethnicity, BMI, or comorbidities that they can consider including in these models?

*Unfortunately we do not have this information.*

Table 1: Did the authors consider treating age as a continuous variable?

*Age was included in groups in the model as these were the groups that we used for the stratification.*

*We decided to use these age categories because they were based on the groups used in the mRNA-1273 and BNT162b2 trials (ref 1 and 2). Additionally, the percentage of participants in these groups were based on the percentage of PLWH within those categories in the Netherlands, as now elaborated in the methods.* Page 9, lines 167-171

Did the authors consider including days between doses as a covariate in the models?

*As mentioned in table 1, the median days between vaccinations were 35 with interquartile range of 35-36. As this range was so small, we did not think adding days between doses would have any discriminative power.*

Can the authors also please present the fully adjusted models in the tables and figures?

*The models have been added to the supplemental data.* Supplemental figures S3-S8

**Reviewer #2:**

Hensley et al. have performed a large-scale study of people living with HIV (PLWH) and their response to a variety of covid19 vaccines. The size of the study is a major strength. The clearest findings from the manuscript are a lower binding antibody response in PLWH compared to controls and a large alteration in binding titres dependent on vaccine type. They do not evaluate antibody neutralisation activity (understandably given the number of samples) nor attempt to relate their binding data to previous studies exploring the relationship between binding/neutralisation titres and vaccine efficacy. Greater discussion of how these binding data fit in the wider cannon of Covid19 vaccine studies (including but not limited to those with PLWH) is needed to make this manuscript of publication quality, please see individual critiques.

The authors do assess cellular immune responses in a sub-group of individuals. However, this data is not particularly clear as the negative control stimulation (an MS associated peptide pool) appears to give a very high response in PLWH. This portion of the data as it currently stands is very confusing and not particularly informative, please see queries below.

Individual critiques

Line 58: The authors state that "the primary endpoint of their study was the anti-spike SARS-CoV-2 IgG response after mRNA vaccination". This is perfectly reasonable, but please acknowledge clearly in the manuscript that neutralisation titres, not just total IgG binding titre, are likely important for vaccine efficacy and that neutralisation hasn't been measured here

*We have added this suggestion to the discussion to elaborate on why we did not perform neutralization assays.* Page 25, lines 456-462

Line 67-67: Please make it clear if any participants don't seroconvert and what % this is.

*We have added the number of participants receiving an mRNA vaccine that have a response over 300 BAU/ml (which we have defined as an adequate response), as well as the participants that were on the limit of detection.* Page 4, line 78-79

Line 76-77: "To reach and maintain the same serological responses and vaccine efficacy as HIV-negative controls, additional vaccinations are probably required."

This statement needs to be properly caveated as this study has not directly addressed vaccine efficacy nor explored links between the titres measured/serological differences observed and vaccine efficacy.

*We have removed some words to more accurately reflect the results shown in our manuscript based on this reviewer’s suggestion.* Page 5, line 92

Line 87-89: "After the SARS-CoV-2 vaccine registration trials, vaccinations were rolled out globally. However, people with immune deficiencies were only sporadically included in the original phase three SARS-CoV-2 vaccination trials."

Please add details of PLWH included - numbers / % as appropriate

*We have removed this paragraph to focus more on PLWH than people with other immune deficiencies.*

Line 89-91: "Subsequent reports indicated markedly diminished responses in solid organ transplant recipients, and lower responses in haemodialysis patients, stem cell recipients or patient groups on specific immunosuppressant drugs for immune disorders [6-9]."

Please include the magnitudes of these lower responses to put your findings here into context.

*We have removed this paragraph to focus more on PLWH than people with other immune deficiencies because it becomes increasingly clear that there are substantial differences between the different causes of immunoddeficiency.*

Line 97-98: "that after SARS-CoV-2 infection, lower IgG concentrations and neutralising antibody titres were found in PLWH compared to controls [13]"

There are other studies where this was not found, and these should also be cited.

*We queried Pubmed and Medxriv using the search terms ‘hiv’ and ‘sars-cov-2’ but were unable to find any other studies that would fit the profile the reviewer is pointing at.*

Line 103-4: "A good understanding of vaccination response in PLWH becomes even more important now that variants of concern (VOC) continue to arise and partially escape vaccine induced immunity [19]."

Should be mentioned that persistent infection in PLWH is also a plausible mechanism for generation of VOC (there are at least 2 papers/pre-prints from South African groups on this)

*This indeed a possible important mechanism for VOC, we have added this suggestion.* Page 7, line 153-154

Line 138: "validated IgG Trimeric chemiluminescence immunoassay (DiaSorin Liaison)"

Please provide information on how this assay performs with either of the WHO standards or how results can be converted?

*We have added a reference that shows the performance of the assay.*

*Unfortunately, there is currently no method to convert results obtained from different methods. (Infantino, Maria et al. “The WHO International Standard for COVID-19 serological tests: towards harmonization of anti-spike assays.” International immunopharmacology vol. 100 (2021): 108095. doi:10.1016/j.intimp.2021.108095)*

*Saker K, Escuret V, Pitiot V, Massardier-Pilonchéry A, Paul S, Mokdad B, Langlois-Jacques C, Rabilloud M, Goncalves D, Fabien N, Guibert N, Fassier JB, Bal A, Trouillet-Assant S, Trabaud MA. Evaluation of Commercial Anti-SARS-CoV-2 Antibody Assays and Comparison of Standardized Titers in Vaccinated Health Care Workers. J Clin Microbiol. 2022 Jan 19;60(1):e0174621. doi: 10.1128/JCM.01746-21. Epub 2021 Oct 27. PMID: 34705539; PMCID: PMC8769743.* Page 10, line 203

Line 147: Please define MOG at first use.

*The definition has been added.* Page 11, line 212-215

Line 155 & 159: Please change height to magnitude.

*This has been changed.* Page 11, line 223 and 227

Line 161: "hyporesponse as 50-300 BAU/mL and non-response as <50 BAU/mL, based on previous studies"

If non-response equates to seronegative, please use that term.

*We defined a clinically relevant non-response at 50 BAU/mL based on the manufacturers cut-off of 33.8 BAU/mL and clinical expertise. The lower limit of detection of the assay was 44.81, we have now defined these more accurately and mentioned the participants in these categories.* Page 11, line 2228-232

Line 204: "At sampling before vaccination, 53 (4.2%) PLWH had antibodies against SARS-CoV-2 above test cut-off and were excluded."

Please state if these were antibodies against N or S?

*This has been specified.* Page 13, line 276

Line 234: "In all vaccines investigated, antibody concentrations were lower in PLWH compared to controls (Fig 2)."

Please state this after 2 vsaccine doses

*The sentence has been elaborated to contain ‘after completion of the vaccination schedule’. As the Janssen vaccine schedule consisted of only one vaccine.* Page 16, line 305

Fig 2: Antibody concentration in PLWH and controls after vaccination The results in this figure are intriguing and given the high numbers of samples tested please comment on whether this study is adequately powered to say which vaccine PLWH should have given the defect observed between vaccine types.

*Our recommendation has been added to the discussion.* Page 25, line 481-482

Line 254: "We found no association between nadir CD4+ T-cell count and antibody responses"

Since Ab response in PLWH have such a different spread especially after bnt162b2, please make a correlation plot to visualize what kind of clinical parameters the people with Ab <300BAU/ml. Even if there is no correlation, seeing the data this way would be informative.

*We performed a multivariate model that adjusts for the variables which are present in our dataset and are associated with antibody response (such as sex, the different age groups and CD4+ T cell counts). Therefore we do not believe that a univariate correlation will be of additional value, as it does not take all the variables into account.*

Line 259: "over 500 cells/μL (2.936 95%CI 1.961-4.394) (both 259 p<0.001) with increased antibody concentrations of 184.34% and 193.59% respectively."

Increased compared to what? people with CD4 count under 200? Please clarify.

*This has been clarified.* Page 17, line 333

Line 266: please call "non-response" seronegative unless there is some reason to believe this assay has an artificially high limit of detection

*The assay has a cut-off value of 33.8 BAU/mL with a lower limit of detection at 4.81 BAU/mL. Based on this value and clinical expertise we defined a non-response in our cohort as under 50 BAU/mL. We have highlighted these differences throughout the text.*

Line 273-6: "Being 56 to 65 years of age (p=0.025, OR 2.919) and most recent CD4+ T-cell count between 250 and 500 cells/μL or over 500 cells/μL (both p<0.001 OR 7.810 and 15.853 respectively) were significantly associated with an antibody response of more than 50 BAU/mL. Sex, viral load and nadir CD4+ T-cell count were not associated."

What about associations with being off treatment/ blips/ poor adherence or treatment type?

*Unfortunately we do not have information on therapy adherence or blips.*

Line 285:

Firstly, it should be clearly explained here why nucleocapsid responses are looked at and why MOG is used as a negative control.

*We have elaborated now in the manuscript on why nucleocapsid responses are looked at and why the use of MOG as a negative control rather than DMSO/media stimulation was done.* Page 11, line 212-215

Secondly, the MOG responses in FigS3 are almost as big as the responses shown in S3 thus the subtraction does not make much sense. There seems to be some technical issue that has not been well described here so it's impossible to assess the value of these data. If the S3 is accurate then there is a negative control MOG response of equivalent magnitude to the Spike specific response, both before AND after vaccination. This doesn't make sense. If PLWH have an oddly higher reactivity against MOG then unstimulated control would be a better comparator. Was an unstimulated control performed? Can this data be included used to perform the subtraction instead?

*The DMSO/media stimulation has been added to figure S4 (was S3) S3. As in the MOG stimulation, a lot of background/a-specific reaction can be seen. The results are similar in MOG and DMSO/media and the magnitude of the responses are similar in the same patients.* Figure S4

Fig 3: Cellular immune responses against SARS-CoV-2 in subgroup participants (PLWH) There's no real indication in the data in this figure that cellular responses are worse in PLWH as claimed in conclusion

*In our results we say ‘both CD4+ and CD8+ T-cell responses were of similar magnitude between PLWH and HIV negative individuals.’ And conclude in the discussion ‘. There were still a relevant proportion of PLWH in whom T-cell responses after stimulation could not be measured. However, in the AIM assay this was also observed in controls.’*

*Our overall conclusion is based on the antibody data, not the T cell data.*

Fig 3.A It would be clearer to have one graph for mRNA vaccines and one graph for vectored vaccines. Please indicate how many people have a neg response at T0 and T2. Consider replacing T0/T2 nomenclature by actual baseline/post 2 doses as this is more intuitive for the reader.

*We have added the separate vector and mRNA graphs to the supplemental but believe it to be more informative when bundled due to the limited number of participants in the sub group receiving the vector vaccines. The negative response numbers have been added to the legend. We have changed T0 to ‘Pre’, T1 to ‘Inter’ and T2 to ‘Post’ to make it more intuitive.* Figure S3A and S3B have been added

Fig 3.B Why in the left-hand panel isn't the delta response increasing with vaccination? this is whole spike peptide pool. Data so far from others shows no real alteration in WT vs VOC T cell ELISpot unless just a mutation specific pool is used. So, this seems wrong.

*This data is from the activation induced marker assay (AIM) and stimulation in the ‘delta’ condition is a peptide pool covering the entire S-protein of the B.1.617.2 (delta) variant*

Line 340: "Limited data exist on SARS-CoV-2 vaccination responses in PLWH."

This is true, but there is some data in publicly available studies, and these should be cited and mentioned here to put this work into context

*We elaborate on these results in the second paragraph of the discussion in the context of other studies.* Page 23, lines 426-431

Line 341: "lower SARS-CoV-2 S1-specific IgG levels in PLWH compared to controls" Should add "as measured by diasorin assay" it's important to stress how this result was found as it is different to other studies to date.

*We have added this.* Page 23, lines 417-418

Line 345-6: "Our primary result analysis in participants receiving mRNA vaccines contrasts with most of the small cohort studies performed in PLWH where the authors found similar responses as in controls [16-18]."

This is a major finding and so the authors should expand further on why this difference is found.

*We discuss all the differences found in both mRNA and vector vaccines combined. This has now been clarified.* Page 23, lines 426-431

Line 350: "Most, if not all, of the previous studies had not been powered to detect a predefined size of the impact of HIV on vaccination response"

Again, here there needs to be a discussion around the fact that binding not being the be all and end all of serology and that neutralisation has not been assessed.

*We have added to the discussion to elaborate on why we did not perform neutralization assays.*

Page 25, lines 468-474

Line 352: "Other reasons that may explain why some of the previous studies did not find a lower response in PLWH may be the use of qualitative rather than quantitative antibody responses"

Please expand on why these results are quantitative whereas prior studies are qualitative - this doesn't seem correct (or at least intuitive) to this reviewer.

*We have expanded on our definition of quantitative and qualitative.* Page 23 line 430-431

Line 355: "However, this was observed in other vaccination studies in PLWH previously [23]" Please highlight you mean against other pathogens

Please discuss in text whether this means you are seeing these differences because cohort of PLWH contained more old people compared to other cohorts?

*This sentence related to the difference in sex found in our study, this has been changed to state this more clearly.* Page 23, line 433-435

Line 363: "We observed a relevant IFN-γ production in the negative control stimulation in the PLWH group."

I don't understand this sentence and I don't see the data supporting this in the figures.

*We have added more information to this paragraph to explain the results.* Page 24, lines 442-445

Line 384: "However, at least in HIV-negative people, this can be overruled by a booster vaccination [21]."

Overruled is not the right word for this context… and can a booster fix everything? I think there needs to be more discussion about what these differences might mean for clinical vaccine efficacy and whether extra boosters or higher doses/altered regimen is needed (as per some other vaccines in PLWH)

*We have elaborated the discussion and underlined the importance with new literature published.* Page 25, lines 487-488

**Reviewer #3:**

In this work, Hensley et al. present the data from over 1154 people living with HIV/AIDS (PLWHA) and 884 controls who were vaccinated for Covid-19 to explore the anitgen-specific humoral and celluar immune responses. This is a multi-site study, involving 22 out of 24 centers providing care to PLWHA in The Netherlands.

Their findings line up with findings from other groups, suggesting that the vaccines are immunogenic and well tolerated in PLWHA. They also expand to a more detailed data on the celluar immune response, which is a strong point, despite the number of analyzed subjects.

The results adds, in a compreehensive observation, the need to vaccinate PLWHA for Covid-19. Although no efficacy data is presented and one still lack the marker of protection for the disease, this is important information to be generated and communicated.

Minor comment:

1. Authors should acknowledge findings with other vaccine platforms, including inactivated virus

*We thank the reviewer for their comments. Whilst we believe inactivated vaccines are important, we believe they are currently outside the scope of this research as they are currently not approved for use.*

Again we would like to thank you for your time and consideration in reviewing our manuscript.

Sincerely,

Dr. Casper Rokx, MD PhD

Internist and Infectious Diseases Specialist

Room Rg530, Erasmus University Medical Center, Rotterdam, the Netherlands

Tel. +31681336328‬

[c.rokx@erasmusmc.nl](mailto:c.rokx@erasmusmc.nl)

Kathryn Hensley, MD.

Room Na2107, Erasmus University Medical Center, Rotterdam, the Netherlands

Tel. +31650032249

[k.hensley@erasmusmc.nl](mailto:c.rokx@erasmusmc.nl)
