# Supplementary material for: Immunogenicity and reactogenicity of SARS-CoV-2 vaccines in people living with HIV in the Netherlands: A nationwide prospective cohort study
Source: PLoS Med. 2022 Oct 27;19(10):e1003979. doi: 10.1371/journal.pmed.1003979 (PMC9612532; doi:10.1371/journal.pmed.1003979)
Supplement: Attachment — Submitted filename: Response to reviewers - second revision.docx [file pmed.1003979.s018.docx]

Rotterdam, 19 August 2022

Dear Dr. Turner, Dr. Bosurgi,

We would again like to thank the editorial board of PLoS Medicine and the specialist reviewers for their valuable suggestions and time to review our work, allowing us another opportunity to improve our manuscript. Please find below the point-by-point replies to the questions that were raised, with our answers in italics and the location of the changes in the marked-up copy of the manuscript underlined.

**Comments from Academic editor**

Just noting that in the legend of Fig 2 the authors need to state the p values (and test used) for comparisons between HIV- and PLWH, and how many subjects in each group (they have included this information in the Figure 3 legend). And also state what the numbers above the dot-plots represent.

*We would like to thank the academic editor for this comment and apologise for not seeing this earlier. We have added the missing information to the legend of figure 2; namely the numbers of participants and that the bars represent the geometric mean concentration. No univariate statistical analyses were performed on these data, therefore no p-values and tests were added (Fig 2 (legend) Page 16, line 313-316).*

**Comments from the reviewers:**

**Reviewer #1:**

Comment: Many thanks to the authors for satisfactorily considering and responding to each comment in turn, amending the manuscript where necessary.

*Answer: We would like to thank reviewer #1 for the comments which allowed for us to revise and improve our manuscript.*

**Reviewer #2:**

Comment: The major original critique was that the T cell component was confusing. This has not been well-addressed. It is still unclear if the spike SFU numbers shown in FigS4 have already had the background subtracted or not, I would strongly suggest DMSO subtraction is more comparable to other studies than MOG subtraction. The data in FigS3 look as expected but it doesn't make sense how they are derived from the data in FigS4 as the Spike responses are equal in magnitude to the DMSO/MOG/NC in Fig4S? And the DMSO responses are abnormally large. This is very different to other studies where spike-specific responses are much larger than DMSO - this is how we know the assay has actually worked when running them. Based on the data presented I cannot support their publication as I am concerned there is an artefact, in my view these data should be removed as the serology is valid independently.

*Answer: The reviewer indicated* “It is still unclear if the spike SFU numbers shown in FigS4 have already had the background subtracted or not*”. We apologise for not being sufficiently clear. Figure S4 shows the raw data for the IFN-γ SFC, with the negative controls, MOG and media-DMSO, and the positive control, CEFX, included. We have now added “raw data” to the legend to clarify this.*

*The reviewer* “would strongly suggest DMSO subtraction is more comparable to other studies than MOG subtraction”. *We are using MOG as a negative control as in our experience, this is a more relevant control than media + DMSO for non-specific, spontaneous T cell responses. As suggested by the reviewer, we have now added as Fig S3C the vaccination-mediated increased in S-specific T cells after subtracting the media-DMSO background from the spike-stimulated SFC. Both figures, Fig 3A (MOG subtraction and Fig S3C (DMSO-media subtraction) show similar results, with a significant increase in S-specific T cells after vaccination of PLWH.*

*We disagree with the statement of the reviewer that* “it doesn't make sense how they are derived from the data in Fig S4 as the Spike responses are equal in magnitude to the DMSO/MOG/NC in Fig4S”. *Fig S4 indeed shows a nearly equal IFN-γ SPC for SP1+SP2, MOG, and media-DMSO for stimulation of PBMCs collected pre-vaccination. However, this is expected since these PLWH were not previously infected with SARS-CoV-2 and therefore should not have a specific T cell response to the spike peptide pool. Post-vaccination, a very clear increase was detected for SP1+SP2 after MOG subtraction (from median 27.5 SFC pre-vaccination to median 152.5 SFC per million PBMCs post-vaccination (Page 18, line 356). This is also clearly visible in Fig S4 when column 2 (SP1+SP-2 Post) is compared with column 6 (MOG post) or column 9 (Media + DMSO post). The misinterpretation of this data by the reviewer could be due to the fact we did not accurately indicate on the y axis that this is a log10 scale. We apologise for this and have corrected it in the new version. We would like to point out to the reviewer that the fold increase of Spike-specific IFN-γ SPC from pre- to post-vaccination is 28 (see figure included in this reply), which also clearly shows that indeed there is a difference between pre- and post-vaccination S-specific T cell responses.*

*The reviewer also states the following:* “And the DMSO responses are abnormally large. This is very different to other studies where spike-specific responses are much larger than DMSO” *We certainly agree with the reviewer that the number of IFN-γ-producing cells after MOG (or media-DMSO) is higher when using PBMCs from PLWH than what we, and others, have seen when using PBMCs from HIV-uninfected people. We do not know whether the reviewer specifically refers to his or her own experience with PBMCs from PLWH or PBMCs from HIV-uninfected individuals. In the figure added to this reply, we show the IFN-γ SFC we received from HIV-uninfected people pre- and post-vaccination using the same peptide pools and ELISpot assays, indicating also a lower spontaneous IFN-γ release of cells from healthy individuals in the presence of media-DMSO or MOG compared to PBMCs from PLWH. For the PBMCs from HIV-negative participants, the median IFN-γ SPC/1 Mill PBMCs for MOG before and after vaccination is 13 and 10, respectively and the median IFN-γ SPC/1 Mill PBMCs for media-DMSO before and after vaccination is 5 and 7, respectively. For PBMCs from PLWH, the median IFN-γ SPC/1 Mill PBMCs for MOG is 255 pre-vaccination and 203 post-vaccination, and the median IFN-γ SPC/1 Mill PBMCs for media-DMSO is 260 pre- and 223 post-vaccination, respectively. Unfortunately, we could not find published raw data from ELISpots using PBMCs from PLWH to support our observation.*

*We stated in the revised manuscript that this heightened IFN-γ-response could be due to the increased activation status of immune cells from PLWH (Page 23, line 441-443). We have now further emphasized this by including a reference (PMID: 29974672) which shows that immune activation leads to higher background spots in ELISpot assays of HIV-negative individuals (Page 23, line 443-445). This background correlates with activated immune cells, such as CD38+HLA-DR+ T cells, cell populations which are increased in PLWH.*

*The reviewer stated that* “This is very different to other studies where spike-specific responses are much larger than DMSO - this is how we know the assay has actually worked when running them.” *We politely disagree with this statement. We are using the ELISpot assay to answer the question whether these patients have IFN-γ-response to SARS-CoV-2 Spike peptides. Before vaccination and without infection, these PLWH did not show a greater response when stimulated with the Spike peptide pool then in the presence of MOG and media-DMSO. However, this does not indicate that the assay did not work but indicates that these participants indeed did not come in contact with the virus or the vaccine. We have included, and shown in Fig S4, CEFX as a positive control to ensure the quality of the PBMCs and the functionality of the assay. As shown in FigS4, all participants showed a response to CEFX pre-vaccination, which, as expected, also did not change after the SARS-CoV-2-specific vaccination.*

*The AIM data (Fig 5) show as well an increase in activated CD4+ T cells after vaccination, further supporting our conclusion that vaccination increases S-specific T cells.*

*We therefore hope that the reviewer now agrees with us that the presented data showing increased spike-specific T cell response after vaccination as shown by IFN-γ-secreting cells is not an “*artefact*” but suggests that vaccination of PLWH increases the frequency of SARS-CoV-2 Spike-specific T cells.*

Comment: The issue of referring to a non-response rather than seronegative has not been resolved, I think this would be clearer for the wider community doing a variety of assays not just this specific one. Also, they refer to clinical expertise in drawing the line at 50 BAU/ml where the manufacturer says 33.8 BAU/ml. I understand views on what is a "useful" response may be taken in a clinical setting but it doesn't make clear sense to me in a research paper to disregard manufacturers guidelines on cut-offs based on sensitivity and specificity data using known positive and negative cases that enabled clinical use of the assay in the first place and call this "non-response". Moreover, the term "adequate response" for >300 BAU/ml is misleading as it implies some knowledge of how this assay relates to protection which has not been explored here. I would strongly suggest using the term "normal" rather than "adequate".

*In the initial study protocol, which was set up at the beginning of the national vaccination campaign, we defined the cut-of value at 50 BAU/mL for non-responders with virologic experts in the Netherlands. We understand that this is confusing as the reviewer states ‘*they refer to clinical expertise in drawing the line at 50 BAU/ml where the manufacturer says 33.8 BAU/ml’. *As per suggestion, to remove any confusion and to be able to compare this data to other research, we* *have therefore changed the cut-off from 50 BAU/mL to 33.8 BAU/mL as per the manufacturers guidelines. With changing the positivity cut-off, we have also addressed ‘*The issue of referring to a non-response rather than seronegative has not been resolved’. *We now refer to the presence or absence of an antibody response when discussing the cut-off of 33.8 BAU/mL. (Page 9, line 195; Page 10, line 224-229; Page 12, line 255; Fig 2 (including legend Page 16, line 320); Page 17 line 340-341; Page 18 line 348-352)*

*In addition, the reviewer states* *‘*I think this would be clearer for the wider community doing a variety of assays not just this specific one’. *We agree with the reviewer that performing other assays is always a good addition to increase the validity of our research, one example discussed in the manuscript is the neutralisation assay (Page 24, line 267-473). As discussed in the manuscript and also stated in our first response to the editor we did not include these as ‘neutralisation correlated with magnitude of antibody response. Whilst we believe that this could indeed be of additional value, considering the reasons given, and the time and manpower necessary to perform these tests, and also the importance of our message to be released as soon as possible’.*

*The reviewer states that ‘* the term "adequate response" for >300 BAU/ml is misleading as it implies some knowledge of how this assay relates to protection which has not been explored here*’. We do not agree with this reviewer in saying that this term is ‘*misleading’. *This cut-off value was known to correlate with a neutralising capacity of 1:40, which was defined as minimally protective, as stated In our manuscript (Page 11, line 226-228). The term ‘adequate’ for participants with a response over 300 BAU/ml is a term which has been used in other COVID-19 studies performed in the same research programme, and we have therefore chosen to remain consistent with other research. (PMID: 34767759). However, it is important that this research refers to the wild type variant, which was circulating at the time of start of this study and the referred articles. We have therefore specified in the methods that this cut-off is based on the SARS-CoV-2 wild type variant. (Page 11, line 35).*

Comment: The new Author summary also has a major issue in the following claim:

"Because the height of the antibody response correlates with protection against symptomatic infection"

This paper does not look at protection in anyway, so it is not a good idea to make this claim here. If there are papers to support that binding responses (especially this particular assay and the cut offs used by these investigators) correlate with protection from symptomatic infection, then these should be discussed in the discussion and not presented as a key finding /outcome of this piece of work in the author summary. The discussion presents a set of studies linking various parameters to one another but not the direct link of binding to protection, and importantly not the magnitude of binding response in this assay to protection so do not support this statement, and regardless I don't think it belongs in the Author Summary as it is not one of their findings.

*Answer: We agree with the reviewer that ‘*This paper does not look at protection in anyway*’ and it is true that this is not the conclusion of our study, we have therefore been removed this line from the author summary. (Page 6, line 121-122).*

We would like to thank you anew for your time and consideration in reviewing our manuscript.

Sincerely,

Dr. Casper Rokx, MD PhD

Internist and Infectious Diseases Specialist

Room Rg530, Erasmus University Medical Center, Rotterdam, the Netherlands

Tel. +31681336328‬

[c.rokx@erasmusmc.nl](mailto:c.rokx@erasmusmc.nl)

Kathryn Hensley, MD.

Room Na2107, Erasmus University Medical Center, Rotterdam, the Netherlands

Tel. +31650032249

[k.hensley@erasmusmc.nl](mailto:c.rokx@erasmusmc.nl)
