# Supplementary material for: Immunogenicity and reactogenicity of SARS-CoV-2 vaccines in people living with HIV in the Netherlands: A nationwide prospective cohort study
Source: PLoS Med. 2022 Oct 27;19(10):e1003979. doi: 10.1371/journal.pmed.1003979 (PMC9612532; doi:10.1371/journal.pmed.1003979)
Supplement: Attachment — Submitted filename: Response to reviewers - final.docx [file pmed.1003979.s019.docx]

Rotterdam, 19 September 2022

Dear Dr. Turner, Dr. Bosurgi,

We would like to thank the editorial board of PLoS Medicine and the reviewer for their review of our work and the opportunity to publish our results in PLOS Medicine. Please find below the replies to the minor revisions, with our answers in italics and the location of the changes in the marked-up copy of the manuscript underlined.

**Requests from Editors:**

1)	The Data Availability Statement (DAS) requires revision for each data source used in your study. If the data are not freely available, please include an appropriate contact (web or email address) for inquiries (this cannot be a study author).

2)	In the supplementary files, please indicate p <0.001 instead of p = 0.000

*We would like to thank the academic editor for their requests.*

1) *We have updated the Data Availability Statement (DAS) for each data source in the manuscript and in the editorial manager system (line 57-62)*
2) *We have replaced all occurrences of p = 0.000 by p<0.001 (throughout the supplemental data)*

**Comments from the reviewers:**

**Reviewer #2:**

Acceptable clarifications have now been made to the T cell part of the story.

*We would like to thank reviewer #2 for their critical review and are glad that our results are now clear.*

Thank you again for allowing us to share our results in PLOS Medicine.

Sincerely,

Dr. Casper Rokx, MD PhD

Internist and Infectious Diseases Specialist

Room Rg530, Erasmus University Medical Center, Rotterdam, the Netherlands

Tel. +31681336328‬

[c.rokx@erasmusmc.nl](mailto:c.rokx@erasmusmc.nl)

Kathryn Hensley, MD.

Room Na2107, Erasmus University Medical Center, Rotterdam, the Netherlands

Tel. +31650032249

[k.hensley@erasmusmc.nl](mailto:c.rokx@erasmusmc.nl)
